# Femoral Neck System in Pauwell III Femoral Neck Fractures: A Bibliometric Analysis and Review

**DOI:** 10.7759/cureus.104822

**Published:** 2026-03-07

**Authors:** Apoorva Agrawal, Rizwan Khan, Teja P Rongali, Arvind Kumar

**Affiliations:** 1 Orthopedics, Santosh Medical College and Hospital, Ghaziabad, IND; 2 Orthopedics, K G Medident Medical & Dental Care Center, Ghaziabad, IND; 3 Orthopaedics, All India Institute of Medical Sciences, New Delhi, IND; 4 Orthopaedics, Jai Prakash Narayan Apex Trauma Centre (JPNATC) All India Institute of Medical Sciences, New Delhi, IND

**Keywords:** bibliometric analysis, femoral neck fracture, femoral neck system (fns), finite element analysis, internal fixation, pauwels iii

## Abstract

The femoral neck system (FNS) is a relatively new internal fixation device designed to improve stability in unstable femoral neck fractures. Despite its increasing use, a comprehensive mapping of the research landscape regarding its application in high-shear Pauwels III fractures is lacking. This study aims to provide a bibliometric analysis of the current literature to identify trends, research hotspots, and evidence gaps.

A systematic search of the PubMed database was conducted for studies published between 2017 and 2026. Data extraction included publication year, source journals, authorship, institutional affiliations, and country of origin. Bibliometric mapping was performed using the R-package Bibliometrix. Studies were categorized by methodology (Clinical, Biomechanical/Cadaveric, Finite Element Analysis, or Mixed) and level of evidence.

Thirty-five studies were identified, with a mean document age of 2.51 years, reflecting a burgeoning interest in the FNS. Research is heavily concentrated in East Asia, with China contributing 88.5% (n=31) of the total output. *BMC Musculoskeletal Disorders* was the most prolific journal (n=7). The majority of studies utilized computational or experimental designs: 14 FEA, nine biomechanical/cadaveric, and nine clinical studies. Notably, most clinical studies were retrospective cohorts providing Level III evidence, with an absence of Level I or II randomized controlled trials.

The evidence base for the FNS in Pauwels III fractures is rapidly expanding but remains in a foundational phase dominated by biomechanical and finite element simulations. While early clinical results from specialized centers in China are promising, there is a critical need for prospective, multicenter international trials to elevate the level of evidence and validate long-term patient outcomes across diverse populations.

## Introduction and background

Femoral neck fractures have long been described as the “unsolved fracture” because they uniquely combine intracapsular fracture biology, tenuous femoral head vascularity, and high mechanical demands [1]. Despite advances in implant design, surgical timing, and technique, clinically significant rates of nonunion, avascular necrosis, and reoperation persist, even after anatomically accurate fixation [1,2]. No single treatment strategy reliably optimizes outcomes across patient age groups and fracture patterns, making femoral neck fractures one of the few injuries in orthopaedics where failure often reflects biological uncertainty rather than technical inadequacy.

Vertically unstable femoral neck fractures, commonly classified as Pauwels type III, are particularly challenging due to the predominance of shear forces across the fracture site. Multiple cancellous screws are often insufficient to counter these forces and are associated with higher failure rates in such patterns [3]. Consequently, fixed-angle constructs such as the sliding hip screw (SHS), frequently augmented with a derotation screw, horizontal screw, or medial buttress plate, have been advocated for these injuries [3-5]. More recently, the femoral neck system (FNS) has been introduced and adopted clinically as an alternative fixation option, including for unstable and vertical fracture patterns [6].

The FNS is a fixed-angle, minimally invasive internal fixation device developed to address the mechanical challenges of femoral neck fractures, particularly unstable and vertically oriented patterns. The system combines a large central bolt that provides angular stability and controlled dynamic compression with an anti-rotation screw to enhance rotational control of the femoral head fragment. These components are connected to a small lateral locking plate, which anchors the construct to the femoral shaft using one or two locking screws.

The biomechanical principle of the FNS is to function as a low-profile, angular-stable device that resists varus collapse and rotational instability while allowing controlled sliding at the fracture site to promote compression and healing. By integrating fixed-angle stability with dynamic compression in a minimally invasive design, the FNS aims to combine the mechanical advantages of a dynamic hip screw with the reduced surgical footprint of percutaneous screw fixation.

Several comparative analyses suggest that, when compared with traditional implants such as cannulated screws or SHS, the FNS may reduce operative time, improve rotational stability, and lower complication rates, although results remain variable [7]. Despite this, many centers continue to prefer SHS with or without anti-rotation augmentation, or optimized multiple screw constructs, particularly when long-term outcomes are prioritized [2].

Given the relatively recent introduction of the FNS, the body of evidence evaluating its role in unstable femoral neck fractures remains evolving, and clinical consensus regarding its selection over conventional fixation methods has yet to be established. Understanding the nature, volume, and characteristics of the published literature on FNS use in Pauwels type III fractures may assist surgeons in informed implant selection, outcome anticipation, and identification of key contributors within this field. The purpose of this bibliometric analysis is to identify and analyze publications investigating the role of the FNS in Pauwels type III femoral neck fractures.

## Review

Methodology

**Figure 1:**
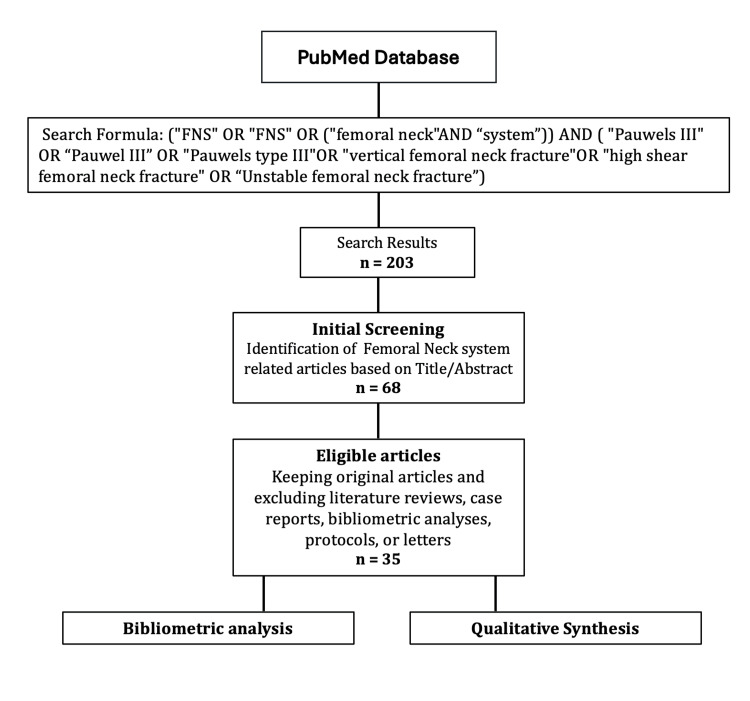
The search strategy of the current analysis of the femoral neck system in Pauwel III femoral neck fractures.

A bibliometric analysis was conducted in accordance with the following structured workflow (Figure 1):

Search Strategy and Initial Screening

A comprehensive electronic search was performed on PubMed by two independent investigators (AK and AA) on February 16, 2026. The search string utilized Boolean operators to capture relevant literature:

 ("FNS" OR "FNS" OR ("femoral neck" AND “system”)) AND ("Pauwels III" OR “Pauwel III” OR "Pauwels type III" OR "vertical femoral neck fracture" OR "high shear femoral neck fracture" OR “Unstable femoral neck fracture”)

Initial screening was performed based on titles and abstracts to identify original research investigating the role of the FNS in femoral neck fractures. Studies were excluded if they were literature reviews, case reports, bibliometric analyses, protocols, or letters to the editor.

Full-Text Eligibility and Refinement

To narrow the focus to high-shear fracture patterns, two authors (AK and RK) performed a secondary full-text review. This phase specifically identified studies investigating FNS in the context of Pauwels Type III, vertical, or high-shear femoral neck fractures. Any discrepancies regarding study inclusion were resolved through consensus and mutual discussion.

Data Extraction and Bibliometric Mapping

Bibliometric data from the final selection were exported and processed using the open-source Bibliometrix R-package [8]. The following parameters were extracted for following analysis: a) year of publication and source journals, b) authorship, affiliated institutions, and country of origin, and c) publication trends. Furthermore, studies were manually categorized into four distinct groups: Biomechanical/Cadaveric, Clinical, Finite Element Analysis, and Mixed/Other. No quantitative outcome synthesis was planned in this study.

Qualitative Synthesis

Finally, a qualitative synthesis was performed by two reviewers (AK and RT). The full texts were analyzed to identify recurring themes, biomechanical outcomes, and clinical takeaway points, providing a comprehensive overview of the current evidence regarding FNS for unstable femoral neck fractures.

Results

A total of 203 records were identified from the database. After title and abstract screening, 68 studies investigating the role of FNS in femoral neck fractures were retained. Of these, 35 studies met the eligibility criteria and were considered for bibliometric analysis and qualitative synthesis (Table 1) [9-43].

**Table 1:** List of studies investigating the role of FNS in Pauwel III femoral neck fractures.

S. No	Title of Study	Year of Publication	Authors	Journal	Type of Study	
1	Biomechanical Evaluation of the FNS in Unstable Pauwels III Femoral Neck Fractures: A Comparison with the Dynamic Hip Screw and Cannulated Screws	2017	Stoffel K et al. [9]	J Orthop Trauma	Biomechanical / Cadaveric	
2	Biomechanical comparison of the FNS versus InterTan nail and three cannulated screws for unstable Pauwels type III femoral neck fracture	2022	Wang Z et al. [10]	Biomed Eng Online	Biomechanical / Cadaveric	
3	Mechanical effects of sagittal variations on Pauwels type III femoral neck fractures treated with FNS(FNS)	2022	Nan C et al. [11]	BMC Musculoskelet Disord	Biomechanical / Cadaveric	
4	Biomechanical comparison of FNS and cannulated screws coupled with medial plate for treating Pauwels III femoral neck fractures	2023	Nan C et al. [12]	Technol Health Care	Biomechanical / Cadaveric	
5	Biomechanical comparison of femoral neck anti-rotation and support system versus FNS for unstable pauwels III femoral neck fractures	2024	Wang T et al. [13]	J Orthop Surg Res	Biomechanical / Cadaveric	
6	Biomechanical evaluation of percutaneous compression plate and FNS in Pauwels type III femoral neck fractures	2024	Xie X et al. [14]	J Orthop Traumatol	Biomechanical / Cadaveric	
7	Biomechanical Stability of FNS for Pauwels Type III Femoral Neck Fractures Based on Different Reduction Quality	2025	Huang D et al. [15]	Z Orthop Unfall	Biomechanical / Cadaveric	
8	[Biomechanical analysis of four internal fixations for Pauwels Ⅲ femoral neck fractures with defects]	2023	Su ZH et al. [16]	Zhongguo Gu Shang	Biomechanical / Cadaveric	
9	Biomechanical evaluation of different internal fixation methods based on finite element analysis for Pauwels type III femoral neck fracture	2022	Jiang X et al. [17]	Injury	Biomechanical / Cadaveric	
10	FNS versus four cannulated compression screws (CCSs) in the treatment of young patients with Pauwels type III femoral neck fracture: a retrospective comparative study	2025	Gao Y et al. [18]	J Orthop Surg Res	Clinical Study	
11	FNS versus cannulated screws with a medial plate for the fixation of Pauwels type III femoral neck fractures in young patients: a retrospective cohort study	2025	Duolikun D et al. [19]	BMC Musculoskelet Disord	Clinical Study	
12	FNS Versus Total Hip Arthroplasty in the Treatment of Pauwels Type III Unstable Femoral Neck Fractures in Patients Aged 60-70 Years: A Comparative Analysis of Clinical Efficacy and Hip Function	2026	Zong X et al. [20]	Orthop Surg	Clinical Study	
13	[Short term follow-up of femoral neck dynamic cross screw system and threaded cannulated screw in the treatment of vertically unstable femoral neck fractures]	2024	Wang Q et al. [21]	Zhongguo Gu Shang	Clinical Study	
14	A short-term efficacy comparison between the FNS and THA as interventions for unstable femoral neck fracture	2025	Si K et al. [22]	Front Surg	Clinical Study	
15	Clinical efficacy of FNS for treatment of unstable femoral neck fractures in young adults	2024	Guo C et al. [23]	J Int Med Res	Clinical Study	
16	Comparison of Early Clinical Results for FNS and Cannulated Screws in the Treatment of Unstable Femoral Neck Fractures	2021	Zhou XQ et al. [24]	Orthop Surg	Clinical Study	
17	Prospective study of FNS fixation combined with enhanced recovery after surgery for the treatment of unstable intracapsular femoral neck fracture	2024	Changbao W et al. [26]	Acta Orthop Belg	Clinical Study	
18	Length-stable fixation reduces femoral neck shortening in unstable femoral neck fractures: A retrospective comparative study of length-stable dynamic hip screw versus FNS fixation	2026	Kang S et al. [25]	J Orthop Surg (Hong Kong)	Clinical Study	
19	Evaluating three internal fixation techniques for Pauwels III femoral neck fractures via finite element analysis	2024	Li N et al. [28]	Sci Rep	Finite Element Analysis	
20	Finite Element Analysis of Six Internal Fixations in the Treatment of Pauwels Type III Femoral Neck Fracture	2024	Sun X et al. [29]	Orthop Surg	Finite Element Analysis	
21	Finite element comparative analysis of three different internal fixation methods in the treatment of Pauwels type III femoral neck fractures	2022	Ma J et al. [30]	BMC Musculoskelet Disord	Finite Element Analysis	
22	Finite element analysis of FNS in the treatment of Pauwels type III femoral neck fracture	2022	Teng Y et al. [32]	Medicine (Baltimore)	Finite Element Analysis	
23	Comparison of FNS and three cannulated cancellous screws in the treatment of vertical femoral neck fractures: clinical observation and finite element analysis	2023	Huang S et al. [33]	Biomed Eng Online	Finite Element Analysis	
24	FNS and cannulated compression screws in the treatment of non-anatomical reduction Pauwels type-III femoral neck fractures: A finite element analysis	2023	Zhong Z et al. [35]	Clin Biomech (Bristol)	Finite Element Analysis	
25	Bio-mechanical effects of FNS versus cannulated screws on treating young patients with Pauwels type III femoral neck fractures: a finite element analysis	2024	Fan X et al. [36]	BMC Musculoskelet Disord	Finite Element Analysis	
26	A finite element analysis of a low-profile FNS of screws in sleeves in a vertical femoral neck fracture model	2024	Sun J et al. [37]	BMC Musculoskelet Disord	Finite Element Analysis	
27	Computational evaluation of the biomechanical effects of position changes in the FNS on Pauwels type III femoral neck fractures: an in silico study	2025	Zhang X et al. [38]	Front Bioeng Biotechnol	Finite Element Analysis	
28	Finite element analysis of the FNS for different placement positions in the fixation of Pauwels type Ⅲ femoral neck fractures	2025	Zhu Y et al. [39]	Injury	Finite Element Analysis	
29	Biomechanical evaluation of FNS versus non-sliding fixation in the treatment of young patients with Pauwels type III femoral neck fractures: a finite element analysis	2025	Gao Y et al. [40]	BMC Musculoskelet Disord	Finite Element Analysis	
30	Additional Screw Added to the FNS Could Enhance the Stability of Pauwel Type III Femoral Neck Fractures: a Finite Element Analysis	2025	Cha Y et al. [27]	Clin Orthop Surg	Finite Element Analysis	
31	Mechanical effects of surgical variations in the FNS on Pauwels type III femoral neck fracture : a finite element analysis	2022	Jung CH et al. [31]	Bone Joint Res	Finite Element Analysis	
32	Pre-sliding of FNS improves fixation stability in pauwels type III femoral neck fracture: a finite element analysis	2023	Cha Y et al. [34]	BMC Musculoskelet Disord	Finite Element Analysis	
33	[Comparison of FNS and inverted triangle cannulated screws fixations in treatment of Pauwels type Ⅲ femoral neck fractures]	2021	Yang J et al. [41]	Zhongguo Xiu Fu Chong Jian Wai Ke Za Zhi	Other / Mixed	
34	FNS vs. four cannulated screws in the treatment of Pauwels III femoral neck fracture	2023	Lin H et al. [42]	J Orthop Sci	Other / Mixed	
35	Is an additional cannulated screw necessary for unstable femoral neck fractures with comminuted posteromedial cortex by FNS fixation? a biomechanical and clinical study	2025	Fan J et al. [43]	Front Bioeng Biotechnol	Other / Mixed	

Bibliometric Characteristics and Growth Trends

**Figure 2:**
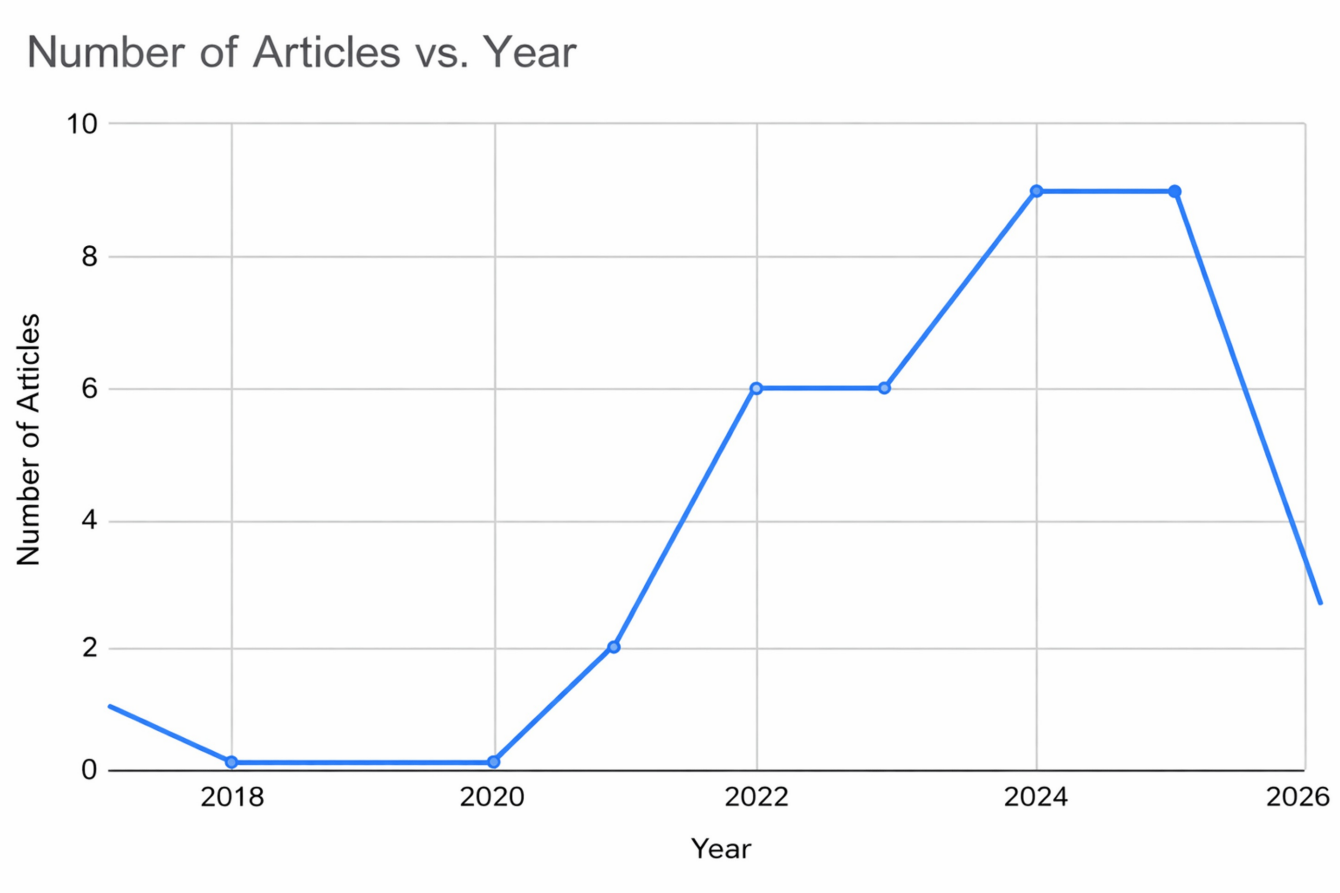
Year-wise contribution of eligible articles.

The included studies were published between 2017 and 2026. This timeframe highlights a rapidly evolving evidence base concerning the FNS in the management of high-shear Pauwels III fractures. The average document age was 2.51 years, underscoring the novelty of the topic. Analysis of authorship revealed a highly collaborative research environment, with 171 authors and an average of 6.09 co-authors per publication; notably, no single-author studies were identified. However, international collaboration remained sparse, accounting for only 2.85% of the total output. The presence of 171 distinct author keywords reflects a relative heterogeneity in the terminology used to describe FNS and unstable fracture patterns. The annual scientific production is detailed in Figure 2.

Source Analysis

**Figure 3:**
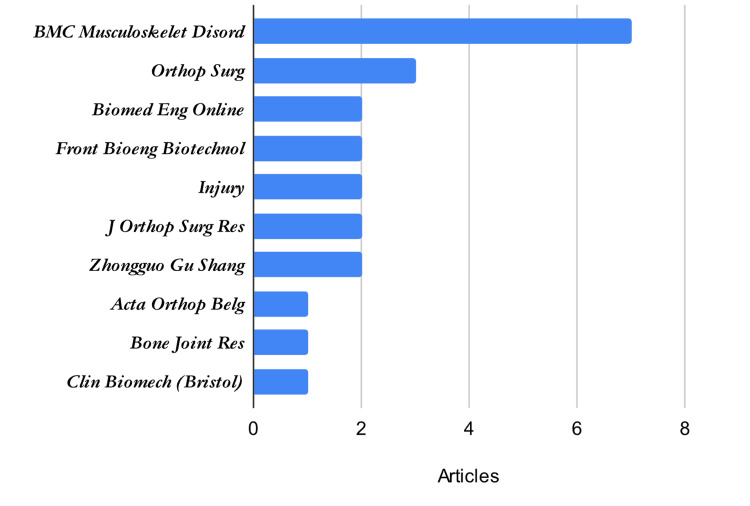
Contribution of different journals toward eligible articles.

The 35 included articles were distributed across 22 peer-reviewed journals. BMC Musculoskeletal Disorders emerged as the most prolific source, contributing 20% (n=7) of the total literature. As illustrated in Figure 3, the remaining publications were distributed among specialized musculoskeletal and biomechanics journals, with the majority of sources contributing only one or two articles. This suggests that while there is a core interest in FNS within specific orthopedic journals, the topic has yet to reach a broad, multi-disciplinary saturation.

Geographical and Institutional Distribution

**Figure 4:**
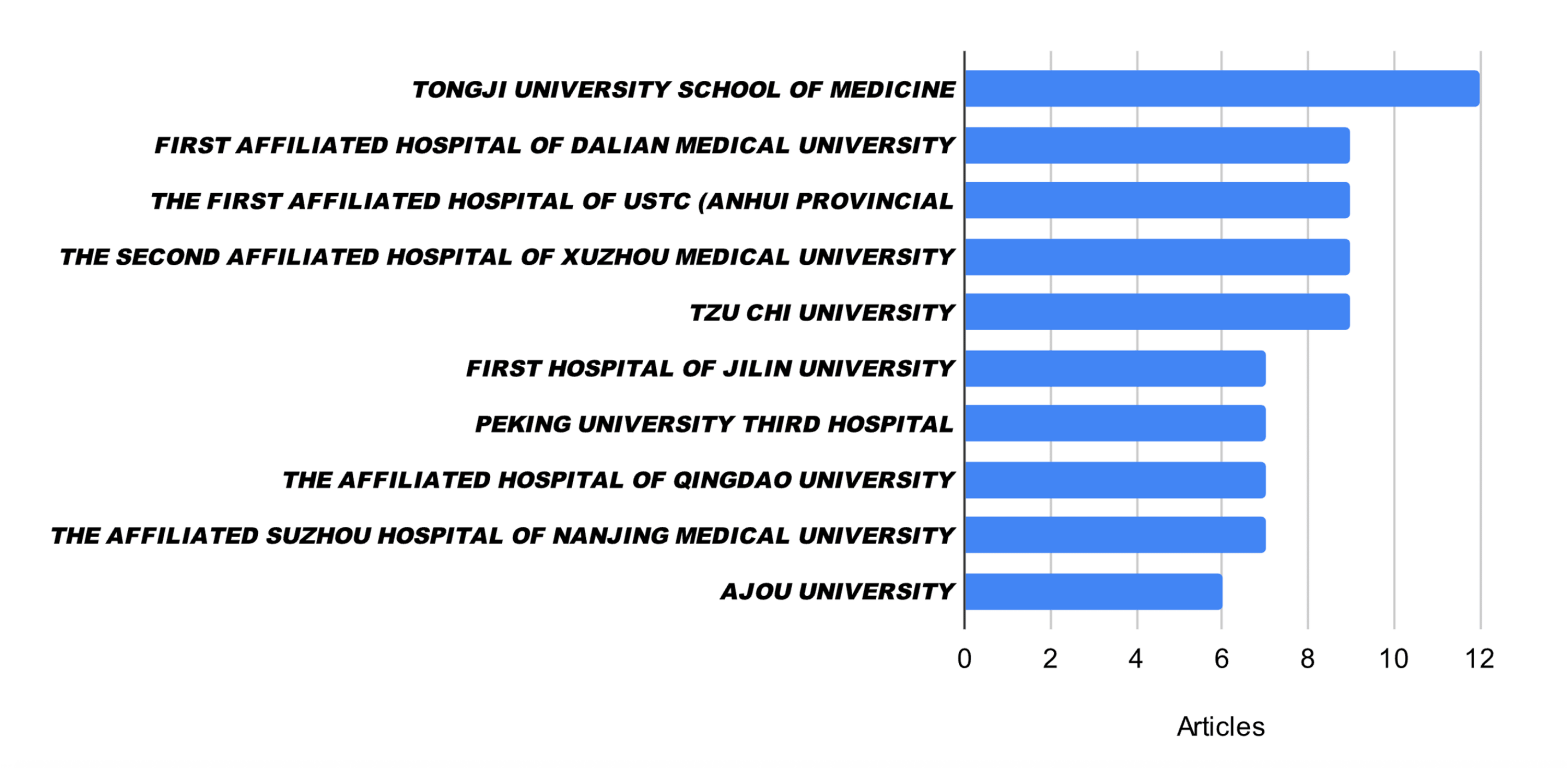
Institutes/hospitals contributing to eligible articles.

The geographic distribution of research was heavily skewed toward East Asia. China demonstrated a clear dominance in this field, accounting for 88.5% of the publications (n=31), followed by South Korea (n=4). Other global regions showed no representation. This regional concentration was also reflected at the institutional level, where the Tongji University School of Medicine led with 12 publications (Figure 4). Overall, the current literature on FNS is primarily driven by a select group of academic centres within China.

Study Design and Level of Evidence

The methodological nature of the included studies varied significantly. Finite Element Analysis was the most frequent study design (n=14), followed by clinical cohorts (n=9) and biomechanical/cadaveric studies (n=9). Additionally, three studies utilized mixed-model methodologies, integrating biomechanical testing with clinical data.

Notably, all but one clinical studies were retrospective cohort designs, corresponding to Level III evidence. To date, no Level I evidence, such as randomized controlled trials or larger prospective comparative studies, has been identified in the literature regarding FNS for Pauwels III fractures.

Discussion

The current bibliometric analysis evaluating the FNS for Pauwels type III or vertically unstable femoral neck fractures demonstrates that this is a nascent yet rapidly evolving research field, with all included publications appearing only in recent years. The literature shows a marked geographic concentration in East Asia, with China and Taiwan accounting for the majority of publications. Although there is substantial collaboration within research groups, international co-authorship remains limited, suggesting that evidence generation is largely regionally driven. Importantly, the current evidence base is dominated by computational and experimental studies, primarily Finite Element Analyses and biomechanical investigations, with relatively sparse clinical evidence.

Biomechanical and Finite Element Evidence

This bibliometric analysis highlights that the current evidence supporting FNS in Pauwels type III femoral neck fractures is predominantly derived from biomechanical experiments and Finite Element Analyses, with relatively limited clinical validation [9-17,28-41]. These studies consistently demonstrate that traditional three-cannulated screw constructs are insufficient for the high-shear environment characteristic of vertical fracture patterns [3,9,10,12,18-20,22,25,34,36,37,41-43]. Although cannulated screws are minimally invasive, they lack the angular and rotational stability required for Pauwels III injuries.

The FNS provides angular and rotational stability comparable to the SHS while requiring a smaller surgical exposure [18-27]. Multiple studies show that FNS is superior to cannulated screws in resisting femoral neck shortening and varus collapse, with two-hole FNS constructs offering greater stability than single-hole designs [15]. SHS augmented with an anti-rotation screw (SHS+ARS) demonstrates improved stability over SHS alone but remains more invasive than FNS. Intramedullary fixation devices, such as the InterTan nail, exhibit the highest axial and bending stiffness; however, their clinical use in femoral neck fractures is limited due to challenges in achieving precise reduction and concerns regarding vascular compromise [10].

Several investigations indicate that medial buttress plating significantly enhances stability when used with cannulated screws, although the plate itself may be subjected to high shear stresses and potential fatigue failure [5]. Design modifications, such as the Femoral Neck Anti-rotation and Support System (FNAS) with increased locking angles, demonstrate improved torsional stiffness by providing better calcar support [13]. Both biomechanical and Finite Element Analysis studies emphasize the importance of central bolt placement, minimal tip-apex distance, and high-quality reduction [9-17,28-41]. Negative buttress reductions are consistently associated with increased implant stress and deformation, highlighting the critical role of reduction quality [15]. Although FNS often demonstrates higher internal implant stress, it typically results in lower stress transmission to the fracture site and distal femur, which may be favorable for fracture healing [13].

Clinical Evidence and Implications

Clinical evidence evaluating FNS in Pauwels type III fractures remains limited and is largely based on retrospective cohort studies [18-26]. In patients aged 18-65 years, FNS demonstrates clinical outcomes comparable to multiple cannulated screw constructs, with similar union rates and functional scores at one year [18]. However, cannulated screw constructs are associated with shorter operative times, less blood loss, and lower hospitalization costs [18]. For highly unstable or comminuted fracture patterns, cannulated screws combined with medial plating (CSMP) appear superior to FNS in promoting union and minimizing femoral neck shortening, particularly when comminution, a major risk factor for shortening, is present [19].

In the 60-70-year therapeutic gray zone, FNS represents a reasonable head-preserving option in active patients [20]. While total hip arthroplasty enables earlier weight bearing and superior early function, FNS has been associated with better functional outcomes at one year, preserving native hip anatomy [20]. Nevertheless, because FNS relies on a controlled sliding mechanism that requires adequate cortical support, medial buttress plating should be considered in comminuted Pauwels III fractures to minimize shortening [12,19]. Given that femoral neck shortening of ≥5 mm is strongly associated with inferior functional outcomes, fixation strategies that effectively limit shortening are particularly important in young, high-demand patients [19]. Finally, the higher implant cost of FNS remains a relevant consideration, and in resource-limited settings, optimized multi-screw constructs may represent a more cost-effective alternative [18].

Our analysis reveals a significant geographic imbalance in the literature, with over 80% of the included studies originating from China. This concentration may be attributed to several factors. First, the FNS was designed to address the high failure rates associated with traditional fixation (like cannulated screws) in active patients with high-shear fractures, a demographic that is substantial in rapidly urbanizing regions. Second, the prominent role of institutions like Tongji University suggests a "center of excellence" effect, where early adoption of a new implant leads to a concentrated surge in biomechanical and clinical validation studies. Perhaps the most critical finding for the orthopedic community is the current ceiling of Level III clinical evidence. All but one clinical study identified was retrospective in nature. While these provide valuable insights into early failure rates and surgical techniques, there is a clear "evidence gap" regarding Level I randomized controlled trials. Future research should transition from "how the implant performs in a computer model" to "how it performs compared to the Gold Standard in prospective, multi-center trials."

There are some limitations of this bibliometric analysis. It is limited by database dependency, as only indexed publications were included, potentially excluding relevant gray literature, non-indexed journals, and conference proceedings. The analysis is constrained by the recent introduction of the FNS, resulting in a small number of publications and limited citation maturity, which may underestimate the long-term academic impact of included studies. Most included studies are biomechanical or Finite Element Analyses, with relatively few clinical investigations, limiting the ability of bibliometric metrics to reflect true clinical influence. A future analysis would be needed to better investigate the further evolving evidence with a larger cross-sectional time frame.

## Conclusions

The current bibliometric analysis identifies a rapid and concentrated expansion of literature concerning the FNS for high-shear Pauwels III fractures between 2017 and 2026. The current evidence base is characterized by a strong reliance on computational modeling and biomechanical validation, primarily driven by high-volume academic centers in China. Currently, clinical data is restricted to Level III retrospective studies. While these early reports are promising, suggesting that the FNS provides a stable alternative to traditional fixed-angle devices, the bibliometric data underscores a significant maturity gap between biomechanical theory and high-level clinical proof.
